# Extensively Drug-Resistant Tuberculosis, Central China, 2007–2009

**DOI:** 10.3201/eid1811.120046

**Published:** 2012-11

**Authors:** Dawei Shi, Hui Li, Yuling Zhao, Qiong Jia, Christopher Coulter, Liang Li, Guofeng Zhu

**Affiliations:** Institute of Pathogen Biology of Chinese Academy of Medical Sciences, Beijing, China (D. Shi, Q. Jia, G. Zhu);; Peking Union Medical College, Beijing (D. Shi, Q. Jia, G. Zhu);; National Institute for the Control of Pharmaceutical and Biological Products, Beijing (D. Shi);; Henan Provincial Centers for Disease Control and Prevention, Zhengzhou, China (H. Li, Y. Zhao);; Pathology Queensland, Brisbane, Queensland, Australia (C. Coulter);; and Beijing Tuberculosis and Thoracic Tumor Research Institute, Beijing (L. Li)

**Keywords:** XDR, MDR, tuberculosis and other mycobacteria, tuberculosis, China, extensively drug resistant, multidrug resistant, Mycobacterium tuberculosis

**To the Editor:** Multidrug-resistant (MDR) tuberculosis (TB), defined as TB caused by *Mycobacterium tuberculosis* resistant to isoniazid and rifampin, is threatening global control of TB. The emergence of extensively drug-resistant (XDR) TB, defined as MDR TB resistant to at least 1 quinolone and 1 of 3 injectable second-line drugs (kanamycin, amikacin, or capreomyin), further jeopardizes TB control and prevention.

In the People’s Republic of China, a country in which the economic cost of TB is high, incidence of MDR TB is higher (10%) (*1*) than the average global incidence (4.8%) (*2*). Published reports of XDR TB prevalence indicate that XDR TB is probably an underestimated problem in mainland China (*3*–*7*). China is a geographically large country, and the characteristics of drug resistance of TB might vary among provinces (*2*). Five regional surveys reported drug-resistance patterns of XDR TB in mainland China, and 3 were conducted in eastern China (*3*–*7*). To determine prevalence of XDT TB in central China, we characterized the resistance of MDR *M. tuberculosis* to second-line drugs, specifically identifying XDR strains, in Henan, a major province of central China. Henan Province has the country’s third largest provincial population (94 million) and high rates of drug resistance to any agent (35.5%) (*2*).

The bacterial population retrospectively analyzed in this study has been described (*8*). In brief, from 2007 through 2009, clinical isolates were collected consecutively by the Henan Center for Disease Control and Prevention TB surveillance system and screened for resistance to 4 first-line drugs. Proportion method–based drug susceptibility testing was conducted for the following critical concentrations: isoniazid 0.2 μg/mL, rifampin 40.0 μg/mL, ethambutol 2.0 μg/mL, and streptomycin 4.0 μg/mL. As a result of that study, 150 MDR TB isolates from TB patients were obtained. The genotyping of all MDR isolates was identified by variable number tandem repeat of mycobacterial interspersed repetitive units based on 16 loci with high discriminatory power.

For the study reported here, we performed additional drug susceptibility testing of 4 second-line drugs at the Henan Center for Disease Control and Prevention TB reference laboratory. We used the Löwenstein-Jensen proportion method, recommended by the World Health Organization, according to the following critical drug concentrations: ofloxacin 3.0 μg/mL, kanamycin 30.0 μg/mL, amikacin 30.0 μg/mL, and capreomycin 40.0 μg/mL (*9*).

Susceptibility results for all second-line drugs tested were reported for 143 (95.3%) of 150 MDR *M. tuberculosis* isolates. Among these 143 isolates, 49 (34.3%) were resistant to ofloxacin, 23 (16.1%) to kanamycin, 17 (11.9%) to amikacin, and 25 (17.5%) to capreomycin (Table). All 17 amikacin-resistant isolates were also resistant to kanamycin, and 16 were also resistant to capreomycin. Also among the 143 MDR isolates, 18 (12.6%) showed resistance to ofloxacin and at least 1 second-line injectable drug and were defined as XDR strains. All 18 XDR *M. tuberculosis* isolates were resistant to isoniazid, rifampin, streptomycin, and ofloxacin; 14 (77.8%), 16 (89.9%), 12 (66.7%), and 17 (94.4%) were resistant to ethambutol, kanamycin, amikacin, and caperomycin respectively. Twelve amikacin-resistant XDR isolates were also resistant to kanamycin and capreomycin (Table).

**Table:** Second-line drug resistance patterns for 143 strains of multidrug-resistant tuberculosis, Henan Province, China, 2007–2009*

Drugs	No. (%) strains
INH + RIF	84 (58.7)
INH + RIF + KAN	1 (0.7)
INH + RIF+ CAP	3 (2.1)
INH + RIF + KAN + AMI	1 (0.7)
INH + RIF+ KAN + CAP	1 (0.7)
INH + RIF+ KAN + AMI + CAP	4 (2.8)
INH + RIF+ OFX	31 (21.7)
INH + RIF + OFX + KAN	1 (0.7)
INH + RIF+ OFX + CAP	2 (1.4)
INH + RIF+ OFX + KAN + CAP	3 (2.1)
INH + RIF+ OFX + KAN + AMI + CAP	12 (8.4)
Total	143 (100%)

*INH, isoniazid; RIF, rifampin; KAN, kanamycin; CAP, capreomycin; AMI, amikacin; OFX, ofloxacin.

Genotyping results demonstrated that XDR strains were distributed diversely in the phylogenetic tree, suggesting that these strains evolved independently. Our results indicated that 12.6% of MDR TB isolates from Henan Province meet the definition of XDR TB, which is less than that found by hospital-based studies performed in Shandong (18.7%), Shanghai (30.0%), and Beijing (14.9%) (*3*,*5*,*6*) but higher than that found by 2 other studies conducted in Beijing and Shanghai (6.3% each) (*4*,*7*). The discrepancy between the percentages of XDR TB and MDR TB strains found in these studies might be explained by the probable inclusion of patients who had been previously treated and patients with chronic TB.

Previous studies found high cross-resistance among all 3 second-line injectable drugs in MDR and XDR TB strains (*5*,*10*). Our results support these observations; capreomycin resistance of MDR and XDR strains (17.5% and 94.4%, respectively) in Henan Province were higher than the average levels (10.2%, 62.5%, respectively) reported by a worldwide study (*10*). Pyrazinamide is an essential drug recommended by World Health Organization guidelines for treatment of MDR TB. Among the population with MDR TB that we studied, 10 (76.9%) of 13 XDR isolates were sensitive to pyrazinamide (data not shown), suggesting that pyrazinamide is still an effective first-line anti-TB drug for most XDR TB patients in Henan Province.

We restricted our investigation to 1 province. However, given the average national prevalence of XDR TB (8% of MDR TB) (*1*) and the magnitude of the population of Henan Province, our findings indicate that the prevalence of XDR TB might be higher in central China than previously documented.
